# The relationship between facial attractiveness and perceived guilt across types of crime

**DOI:** 10.1177/17470218231218651

**Published:** 2023-12-21

**Authors:** Robin SS Kramer, Janie-Lea Jarvis, Michaela Green, Alex L Jones

**Affiliations:** 1 School of Psychology, University of Lincoln, Lincoln, UK; 2 School of Psychology & Counselling, The Open University, Milton Keynes, UK; 3 School of Psychology, Swansea University, Swansea, UK

**Keywords:** Face perception, bias, facial attractiveness, defendant, juror decision-making, guilt

## Abstract

Facial first impressions influence jurors in both laboratory experiments and real courtrooms. Often, more attractive defendants are perceived as less guilty and receive more lenient sentences. However, the type of crime under consideration, as well as the ecological validity of the stimuli presented, may determine the nature of this bias. Here, extending previous work, we considered three crime types (robbery, sexual assault, and murder) and utilised short video clips of male defendants, accompanied by real-world crime descriptions. Crucially, we varied attractiveness by presenting a large set of identities, in comparison with the typical use of one “high” and one “low” attractive face. Using null hypothesis significance testing, we found no evidence that either attractiveness or crime type influenced guilt perceptions. Taking a Bayesian perspective, our results provided some evidence that more attractive defendants were rated as less guilty of murder but more guilty of sexual assault, with no bias observed for robbery. Importantly, however, none of these effects had high certainty and all were small in size. By comparing the extremes of attractiveness, we again found inconclusive evidence of any attractiveness effects, with small differences in all cases. The implications for this departure from previous findings are discussed in terms of ecological validity and the need to consider attractiveness as a continuous rather than binary measure.

## Introduction

Forming first impressions based on facial appearance appears to demonstrate many of the characteristics of an automatic process (mandatory—Ritchie et al., 2017; rapid—Willis & Todorov, 2006; non-conscious—Olson & Marshuetz, 2005). As a result, it is perhaps unsurprising that our judgements of others are often biased by these initial impressions. The “halo effect” (Dion et al., 1972), for instance, describes how we apply socially desirable traits indiscriminately to attractive people, resulting in their receiving more help (Benson et al., 1976), earning higher wages (Pfeifer, 2012), and benefitting from more frequent hiring opportunities (López Bóo et al., 2013). Given this tendency to treat people differently based on their facial appearance, researchers have been investigating the problematic notion that jurors in criminal trials might also be affected by such biases.

Considering the facial appearance of real-world defendants, studies have shown that facial trustworthiness may play a role in sentencing outcomes. Prison inmates sentenced to death were perceived as less trustworthy than those who received a life sentence (Wilson & Rule, 2015, 2016; although see Kramer & Gardner, 2020), implying that jurors were influenced by their first impressions of the defendants’ faces. In addition, baby-faced defendants in small claims courts were more likely to win cases involving intentional actions, but less likely to win cases involving negligent actions (Zebrowitz & McDonald, 1991). Further, inmates with more Afrocentric facial features (i.e., those that are typical of African Americans) received harsher criminal sentences, irrespective of their race (Blair et al., 2004). Finally, defendants who were perceived to be more attractive were treated with greater leniency regarding sentencing (Stewart, 1980, 1985), and in general, more attractive people (specifically, women) were less likely to be arrested and convicted (Beaver et al., 2019). As such, it is clear that facial appearance plays a role in the outcomes of real-world criminal cases.

More extensive investigation has been carried out within a laboratory setting, typically featuring participants in the role of mock jurors, to better understand the nature of these biases. Focussing predominantly on perceptions of attractiveness, these studies have provided mixed evidence regarding its effects on judgements of guilt and sentencing. For example, unattractive defendants (typically of a single gender to avoid the possibility of this influencing biases) were perceived to be more guilty when charged with rape (Deitz & Byrnes, 1981; Jacobson, 1981), murder (Coons & Espinoza, 2018), sexual harassment (Castellow et al., 1990), or less serious charges (Darby & Jeffers, 1988; Piehl, 1977). However, in other work, little or no difference was found as a result of attractiveness manipulations with crimes including sexual assault, murder, and robbery (Ahola et al., 2009; Austin et al., 2013; Beckham et al., 2007; McKelvie & Coley, 1993; Winters et al., 2022). To complicate matters further, defendant attractiveness may also interact with other features of the case, including the attractiveness of the plaintiff (Wuensch & Moore, 2004) and whether jurors have a chance to deliberate or not (Patry, 2008). The specific type of crime is also clearly important (for a meta-analysis, see Mazzella & Feingold, 1994). Indeed, the role that attractiveness could play in the crime itself may be a crucial factor—when the offence was attractiveness-related (a swindle), the attractive defendant received more negative treatment (Shechory-Bitton & Zvi, 2015; Sigall & Ostrove, 1975; Wuensch et al., 1993; Yang et al., 2019). This “beauty penalty” is thought to apply when attractive defendants take advantage of their physical appearance and, as a result, deserve harsher sentences (although this pattern is not always apparent; Wuensch et al., 1991).

Perhaps through varying the type of crime considered, evidence to date has shown that greater attractiveness only sometimes results in more lenient judgements or sentencing outcomes (referred to as the attraction-leniency effect). However, it is also worth noting that laboratory-based studies in this field utilise stimuli that do not closely resemble the experiences had by real-world jurors. Typically, researchers have employed static images of faces (often passport-style photos and not taken inside a courtroom) to represent defendants when investigating attractiveness biases (e.g., Abel & Watters, 2005; Ahola et al., 2009; Beckham et al., 2007; Shechory-Bitton & Zvi, 2015; Sigall & Ostrove, 1975; Wareham et al., 2019; Winters et al., 2022; Yang et al., 2019). However, we know that the specific image of the face selected, as well as its background, can affect perceptions (e.g., Elliot et al., 2010; Jenkins et al., 2011), and that attractiveness judgements from static versus dynamic stimuli are related but not equivalent (e.g., Kościński, 2013; Roberts et al., 2009). In the courtroom, jurors are exposed to “live” defendants over a prolonged period of time, so a single, static image may be an oversimplification of this experience.

Another criticism of previous work in this area is that the influence of defendant attractiveness, when manipulated through the use of facial photographs rather than text descriptions, typically involves the comparison of a single pair of “high” and “low” attractiveness facial photographs (e.g., Austin et al., 2013; Beckham et al., 2007; Castellow et al., 1990; Coons & Espinoza, 2018; Jacobson, 1981; Patry, 2008; Piehl, 1977; Shechory-Bitton & Zvi, 2015; Sigall & Ostrove, 1975; Winters et al., 2022; Wuensch et al., 1993; Wuensch & Moore, 2004; Yang et al., 2019). However, attractiveness is a continuous measure, so a simplified comparison of the two extremes may not provide a generalisable pattern of results. In addition, two specific faces chosen to differ in attractiveness are unlikely to be representative of all faces which are high and low in attractiveness, again resulting in findings that may fail to generalise.

To this end, the current study investigated the potential for attractiveness biases when judging the guilt of simulated male defendants. First, we incorporated three types of crime to better understand whether patterns of bias depended on the nature of the crime itself. Second, we utilised short courtroom videos of our defendants, rather than static images, as a step towards more ecologically valid experiences for our mock jurors. Third, we conceptualised attractiveness as a continuum, and featured a range of identities, rather than comparing a single pair of “high” and “low” attractiveness faces. Fourth, we incorporated real-world descriptions of crimes, rather than fictional descriptions created by the researchers themselves (as is often the case; e.g., Ahola et al., 2009), to better represent the charges faced by the defendants in court and heard by the jurors themselves.

Although the evidence is mixed regarding more attractive defendants being perceived as less guilty (see above), we used this finding to inform our analytic strategy and power analysis. However, given multiple differences between our experimental design/stimuli and those of previous studies, we have chosen to take a more exploratory approach in the current work.

## Method

### Participants

One hundred and fourteen volunteers (age *M* = 35.9 years, *SD* = 13.8 years; 78 women; 84% self-reported as White) gave informed onscreen consent before participating in the experiment and were provided with an onscreen debriefing upon completion. Participants were recruited through “word of mouth” and social media advertisements. The data from five additional participants were excluded due to those participants failing one or more attention checks (three) or providing the same response for all 60 trials (two). The number of participants was determined via simulation (see analytic strategy below).

The experiment presented here was approved by the University of Lincoln’s ethics committee (ID 8643) and was carried out in accordance with the provisions of the World Medical Association Declaration of Helsinki.

### Materials

#### Face videos

Sixty White men were selected from videos posted to the Law & Crime Network on YouTube. All identities were giving testimonies in court and comprised a mixture of defendants, witnesses, and experts. In all cases, the men were smartly dressed (e.g., wearing a shirt, jacket, and tie) and there were no visible cues as to which of these roles they appeared under.

For each identity, a continuous 5s segment was selected from the initial YouTube video in which the person was predominantly front-on and speaking for most or all of the time. The video was also cropped to 350 × 350 pixels to include only the head and the top of the shoulders (and the background contained within that frame). These videos were in colour, with the audio information removed.

#### Crime descriptions

Sixty descriptions of crimes were collected from the London Metropolitan Police and Greater Manchester Police websites. These originally appeared as news bulletins describing arrests and/or appealing for further information and were subsequently shortened to contain a brief summary (one or two sentences) of the particular crimes. Specifically, we collected 20 descriptions for each of three types of crime: (1) robbery/burglary (e.g., “This man is being charged with robbery after four counts of carjackings across two days”); (2) rape/sexual assault (e.g., “This man is being charged with the rape of a woman in the early hours behind a newspaper building”); and (3) assault/murder (e.g., “This man is being charged with murder after a man was stabbed and later died in hospital from his injuries”). For all 60 descriptions, care was taken to remove any graphic or overly descriptive details regarding the crimes committed, injuries sustained, etc. In addition, no identifying information was included (e.g., the date of the crime, the location in which it was committed, or the names of any people involved). The lengths of these descriptions (in words) for each type of crime were as follows—robbery/burglary: *M* = 22.2, *SD* = 4.8; rape/sexual assault: *M* = 22.5, *SD* = 3.5; assault/murder: *M* = 24.8, *SD* = 4.4.

### Procedure

The experiment was completed using the Gorilla online testing platform (Anwyl-Irvine et al., 2020). After consent was obtained, participants provided demographic information (age, gender, and ethnicity) through open-ended responses. Participants were then randomly allocated to either the attractiveness ratings task or the guilt ratings task (for details, see below).

For the attractiveness ratings task, participants viewed all 60 face videos, presented in a random order. On each trial, participants were presented with a video, along with the prompt “How attractive is this man?” Self-paced responses were provided using a 0–9 scale (e.g., Kramer & Jones, 2020; Kramer & Pustelnik, 2021), with the video playing on a continuous loop until a response was given. Participants selected a response by moving a slider along a line at the bottom of the screen and then clicked the “Next” button to proceed to the next trial. The current position of the slider (a value from 0 to 9) was displayed onscreen, allowing participants to alter and refine their choices as needed before submitting their responses. Labels were displayed alongside the left (“very unattractive”) and right (“very attractive”) endpoints of the line.

For the guilt ratings, three versions of the task were created. The 60 face videos were initially divided randomly into three subsets. To create each version of the task, the three video subsets were paired with each of the three sets of crime descriptions (20 of each crime type) using a Latin square design. These pairings were originally random but subsequently held constant across participants (due to the limitations imposed by the online platform). For example, Face 1 appeared with either Crime 1 (a robbery/burglary), Crime 21 (a rape/sexual assault), or Crime 41 (an assault/murder), depending on the version of the task. As such, all faces appeared in all crime types across participants.

On each trial, participants were presented with a video and a crime description, along with the prompt “Do you think this man is innocent or guilty of the crime described above?” Self-paced responses were provided using a 0–9 scale, with the video playing on a continuous loop until a response was given. Participants selected a response by moving a slider along a line at the bottom of the screen (as above) and then clicked the “Next” button to proceed to the next trial. Labels were displayed alongside the left (“definitely innocent”) and right (“definitely guilty”) endpoints of the line.

Two attention checks were inserted during both ratings tasks, appearing before the twenty-first and forty-first trials (dividing the task into thirds), given that attentiveness is a common concern when collecting data online (Hauser & Schwarz, 2016). Each of these two trials instructed the participant to respond with either a rating of “2” or “7”. For instance, “Attention Check: Please respond with a rating of ‘2’ to show you’re paying attention” was displayed onscreen. By requiring participants to provide specific responses, we could identify those who were not paying attention.

Participants were randomly allocated to one of the four tasks: the attractiveness ratings (*n* = 31) or one of the three versions of the guilt ratings (*n*s = 27, 27, 29).

### Analytic strategy and power analysis

We analysed our data using linear mixed-effects models. Specifically, we modelled individual trial guilt ratings of the participants allocated to each guilt rating task, using facial attractiveness (averaged across participants in the attractiveness task, giving each face an average attractiveness rating) and its interaction with a categorical predictor—crime type—as fixed effects. Attractiveness averages were *z*-scored, and robbery/burglary was assigned as the reference category. We included random intercepts for participants, accounting for the multiple ratings each participant provided, and both random intercepts and slopes for faces, modelling the variability in baseline guilt ratings for each face, as well as the effect that appearing in different crime types might have.

There is no straightforward method of estimating the number of participants required to detect effects using mixed models since power depends on both the fixed and random effects. However, as we constrained ourselves to 60 face stimuli and a set experimental allocation approach, we held this aspect of our design fixed and used simulation as a method to estimate the number of raters required for a range of effect sizes of interest. Specifically, we set a magnitude and direction on the attractiveness main effect (the relationship between perceived guilt and attractiveness, regardless of crime type). The interaction term was composed of two coefficients—a sexual assault by attractiveness coefficient and a murder by attractiveness coefficient. Either of these coefficients reaching statistical significance would drive an interaction, and we varied the murder by attractiveness predictor, such that increasing attractiveness resulted in a decrease in perceived guilt, for the murder condition.

We trialled candidate sample sizes between 40 and 100 in increments of 20 participants. Within these candidate samples, we estimated the power of the attractiveness main effect by varying the size of the slope between −.2, −.4, and −.9 (i.e., as attractiveness increases by one standard deviation, guilt ratings decrease by the slope amount). Separately, we also varied the assault/murder by attractiveness slope between .05, .10, and .20 (i.e., for every one standard deviation increase in attractiveness, guilt ratings of assault/murder decrease by the value of the slope), holding the attractiveness main effect constant at −.4. These slopes represent a range of plausible and minimally interesting effects. For example, it is improbable that a one standard deviation change in attractiveness would alter guilt ratings by more than one scale point on average, and changes of less than −.2 on the scale would be so small as to be practically null.

Each combination of sample size and coefficient values was repeated 200 times, and the proportion of times the target effects were statistically significant was taken as the estimate of power. While the main effect of attractiveness showed above 95% power at all sample sizes, the interaction showed above 90% power only for the largest effect size (.20) at all levels. We opted to aim for 80 participants as a sample size.

Finally, we planned to investigate any non-significant effects of interest (the attractiveness main effect and the interaction) using Bayesian methods, which can estimate probabilities of hypotheses as opposed to rejecting a null hypothesis (Kruschke & Liddell, 2018). This involved fitting a Bayesian equivalent of the mixed model described above, and we set normal priors centred on zero with a standard deviation of .5 for all fixed effects therein.

## Results

We fitted a linear mixed-effects model to the data using JASP (Love et al., 2019) and submitted the overall model to an analysis of variance (ANOVA). For significance reports, degrees of freedom were estimated using Satterthwaite’s method. We observed no significant main effect of crime type, *F*(2, 56.71) = .16, *p* *=* .850, indicating guilt ratings were similar across crimes. There was also no significant main effect of attractiveness, *F*(1, 58.01) = .14, *p* = .715, indicating no change in guilt ratings with increasing facial attractiveness. Finally, we also observed no significant interaction between these factors, *F*(2, 57.88) = 1.73, *p* = .186. Although we observed no significant effects, we also examined the coefficients of the mixed model as these directly represented the targets of our power analysis simulation. Specifically, the attractiveness coefficient was an order of magnitude smaller than our lowest effect size estimate and in the opposite direction, *b* = .03, *SE* = .103, *t*(58.64) = .286, *p* = .776. However, the interaction coefficient—assault/murder by attractiveness—was in the midpoint of our planned effect sizes, but also in the opposite direction, *b* = −.123, *SE* = .45, *t*(58.53) = .883, *p* = .381.

### Bayesian analysis

Given that we observed no statistically significant effects, and that the point estimates from our initial analyses indicated effects in the opposite direction to our hypothesis, we further investigated our results using a Bayesian approach. This has the distinct advantage of allowing us to quantify evidence for null hypotheses, as well as the range of credible effects given the data (as opposed to a point estimate).

**Figure 1.**
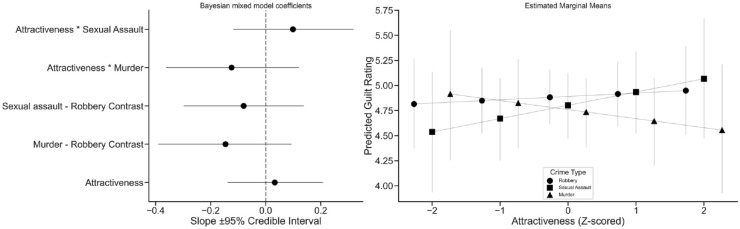
Bayesian model coefficients with 95% credible intervals (left), and the estimated marginal means for guilt ratings under each crime type, at varying levels of attractiveness (right). Error bars are 95% credible intervals.

We first estimated the same mixed-effects model using Bayesian methods and examined the posterior distributions of the coefficients, checking the probability of direction—here, that the effect was positive (Makowski et al., 2019)—and 95% credible intervals. Moreover, we examined the estimated marginal means of the model, predicting guilt scores for each crime type for standardised attractiveness scores at ± 2, ± 1, and zero. These are shown in Figure 1.

The probability of direction and credible intervals indicated, similarly to the frequentist model, that the effect of attractiveness was small but somewhat likely to be positive, *b* = .034, [−.138, .211], *pd* = .651. The main effect components—the coefficients contrasting the crime types of murder and robbery, *b* = −.149, [−.395., .091], *pd* = .113, and sexual assault and robbery, *b* = −.079, [−.293., .135], *pd* = .236, were both negative and likely to be negative, indicating that sexual assault and murder—ignoring attractiveness—received slightly lower guilt ratings. For the interaction components, the attractiveness by murder coefficient was negative and likely to be so, *b* = −.123, [−.362, 0.122], *pd* = .157, suggesting that, compared to the relationship between robbery and attractiveness, attractiveness by murder results in lower guilt ratings (as can be seen in Figure 1). Finally, the attractiveness by sexual assault coefficient was positive, and likely to be so, *b* = .10, [−.118., .317], *pd* = .82, indicating that increasing attractiveness under the sexual assault crime type results in slightly higher guilt ratings, as compared to the same relationship under the robbery crime type. However, none of these effects had high certainty, and were all generally small in actual units, with all credible interval widths being less than half a rating scale point.

As a final analysis, we examined the difference between high and low (± 2 units) attractiveness for each crime type by subtracting the marginal means estimated by the model. We then calculated the probability that this difference was positive (i.e., more attractive people looked more guilty) and also used a Bayes Factor hypothesis test to examine whether each difference lends support to the null hypothesis (the difference was zero) as compared to the alternative hypothesis (the difference was non-zero). In this field, researchers have typically based their conclusions on the comparison of only one or two pairs of “high” and “low” attractiveness faces (e.g., Austin et al., 2013; Beckham et al., 2007; McKelvie & Coley, 1993; Winters et al., 2022). It may be that, at the extremes of attractiveness, a bias in guilt perceptions is evident.

**Figure 2.**
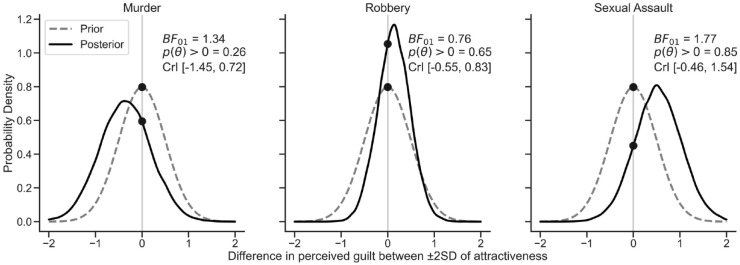
Posterior distribution differences between the estimated marginal means of high and low attractiveness, for each crime type. Points indicate the likelihood of zero under the prior (dashed line) and the posterior (solid line).

We utilised the same prior used in the model estimation, a normal centred on zero with a standard deviation of .5. The results are illustrated in Figure 2. For murder, the average difference was negative, *M* = −.36, [−1.45, .72], *pd* *=* .26. While this indicated that the effect seemed negative, the hypothesis test was relatively inconclusive but indicated the data were more likely under the null, *BF*_01_ = 1.34. For robbery, the result was more uncertain, *M* = .13, [−0.55, .83], *pd* *=* .65, but the hypothesis test reflected somewhat more support for the alternative, *BF*_01_ = .76. Finally, for sexual assault, the difference was larger, *M* = .53 [−.46, 1.55], *pd* *=* .85, and the hypothesis test suggested that the difference was more likely under the null hypothesis, although it did not reach the standard Bayes Factor thresholds, *BF*_01_ = 1.77. Taken together, these results demonstrated that a comparison of the extremes of attractiveness failed to provide any conclusive evidence of a bias in guilt perceptions, with all effects being small.

## Discussion

The current study investigated whether a defendant’s facial attractiveness influenced simulated jurors’ perceptions of guilt across three types of crime—murder, robbery, and sexual assault. To this end, our findings were suggestive but inconclusive.

The traditional, frequentist approach found no significant differences, although it is important to note that our initial assumptions about both the size and direction of the effects were inaccurate. Problematically, this approach is ineffective when faced with the challenge of conclusively demonstrating an absence of difference. For this reason, we also refitted the model using Bayesian estimation, which provided the posterior distribution of the model coefficients. Here, the evidence demonstrated that the effect of attractiveness was, on average, small and somewhat likely to be positive, but with credible intervals showing that it could also be negative (in line with our initial predictions—increasing attractiveness should result in lower guilt perceptions). This approach allowed for probabilistic claims regarding the coefficients, rather than the oversimplified, and to a large extent uninformative, conclusions of non-significance. As Figure 1 illustrated, the overall trends suggested that perceived guilt after a robbery was likely uninfluenced by attractiveness (a mostly flat line), while more attractive defendants looked less guilty for murder, with the reverse being true for sexual assault. Of course, as our analyses reported, the uncertainty in those points was sizable.

To provide some clarity in these results, we considered contrasts between an estimated high versus low attractiveness defendant (±2 *SD*) for each type of crime (see Figure 2). These revealed that, probabilistically, it was about 75% probable that high-attractiveness defendants would be perceived to be less guilty of murder, although the size of the effect was very small. For robbery, judgements of high and low-attractiveness defendants were largely similar. Finally, for sexual assault, these contrasts showed that it was 85% probable that high-attractiveness defendants were perceived as more guilty, although again this effect was small. However, in all cases, the Bayes Factors were inconclusive (the range of .33–3.00). For murder and sexual assault, the Bayes Factors had somewhat more evidence for the null hypothesis, while for robbery the evidence somewhat more favoured the alternative.

Given the typical finding that more attractive people receive lighter sentences (for a meta-analysis, see Mazzella & Feingold, 1994), how might we explain the mixed results presented here? Notable differences between our study and previous work focussed on improvements in ecological validity. To this end, we utilised short video clips of our defendants, within a courtroom context, to better resemble the experience had by real-world jurors. In contrast, previous work has tended to feature static images of faces taken outside of the courtroom (e.g., Abel & Watters, 2005; Shechory-Bitton & Zvi, 2015). This is perhaps surprising since we know that the specific image of the face chosen, along with its background, can alter perceptions (e.g., Elliot et al., 2010; Jenkins et al., 2011), and that attractiveness judgements differ for static versus dynamic stimuli (e.g., Kościński, 2013; Roberts et al., 2009). Here, we also incorporated real-world descriptions of crimes, rather than fictional descriptions created by the researchers (e.g., Ahola et al., 2009), to better represent the charges faced by the defendants in court and heard by the jurors themselves. Of course, regarding both the exposure to the defendant’s face and the information/evidence of the crime itself, we acknowledge that these features of our study only partially recreate actual jurors’ experiences. For example, the likely influence of the defendant’s voice (e.g., Cantone et al., 2019) was purposely absent from the current work to allow us to focus on facial attractiveness specifically. Future studies might aim to incorporate increasingly realistic stimuli to better represent juror experiences.

The use of 5s videos, rather than the static images featured in previous work, likely resulted in participants viewing the faces for longer before making their judgements. Is it possible that this might affect impression formation of itself? Research has shown that forming first impressions happens rapidly, with judgements made after a 100 ms exposure strongly correlating with those made in the absence of time constraints (Willis & Todorov, 2006). As such, we expect that any differences in perceptions as a result of using video rather than photograph presentation would come from the additional information provided by the medium (e.g., how the person moves, speaks, etc.) rather than a simple increase in viewing time. However, further study is needed to answer this question empirically.

As noted above, although the effect sizes were small, our contrasts between an estimated high versus low-attractiveness defendant revealed the clearest results. Perhaps this speaks to the possibly limited influence of attractiveness on perceived guilt. Previous research has typically compared a single pair of faces, pre-selected to represent high versus low attractiveness (e.g., Coons & Espinoza, 2018). Here, we purposely considered a set of faces varying along a continuum of attractiveness (as they do in real-world trials), with our results suggesting little significance overall. However, a comparison of the two extremes of attractiveness supported some (small) influence of attractiveness (although these results remained inconclusive). Therefore, previous results in this domain, typically suggesting that more attractive faces were perceived to be less guilty (e.g., Castellow et al., 1990; Coons & Espinoza, 2018), may be reliant on this comparison of extremes. For defendants in real courtrooms, who tend to fall nearer the average (as most people do, by definition), the influence of attractiveness may not apply. Taken together, we argue that the decision to focus on very limited stimuli, along with tasks designed solely to compare these extremes, might explain the (mixed) evidence of an attractiveness influence in previous work. Further study could consider this experimental issue specifically.

In our work, we investigated three different types of crime to determine whether attractiveness resulted in leniency in all cases or not. Previous work has provided some evidence that different patterns of bias may be evident for different crimes (e.g., Sigall & Ostrove, 1975) and our data certainly appeared to support this conclusion. While assault/murder was suggestive of the typical attraction-leniency effect (i.e., that more attractive defendants were perceived to be less guilty), the opposite pattern was more likely for rape/sexual assault—those men perceived to be more attractive were also judged to be more guilty. This result might be explained through a “beauty penalty” (e.g., Sigall & Ostrove, 1975; Yang et al., 2019), whereby attractive defendants benefitted from their physical appearance and, therefore, deserved harsher sentences. Whether attractive men are, in reality, more likely to be successful when coercing women remains to be seen. Finally, we found little evidence to suggest an influence of attractiveness on guilt perceptions for robbery/burglary, and indeed, this has been mirrored in previous work (e.g., Barnett & Feild, 1978), although it remains unclear as to why this type of crime should differ from our results regarding assault/murder.

Although these patterns and differences were suggested in our data, it was clear that all such effects were considerably smaller than predicted based on previous work. It may be the case, as noted by Austin and colleagues (2013), that the attraction-leniency bias is only apparent for less serious crimes. With crimes that are more serious, attractiveness appears to have less of an effect on sentencing (McKelvie & Coley, 1993; Wuensch et al., 1991). Perhaps in such cases, including the serious crimes featured in the current study, perceptions of guilt are relatively unaffected by attractiveness biases for reasons that have yet to be determined. This idea of crime seriousness represents an interesting avenue for future research.

A notable limitation of the current work was its restriction to the use of White men as supposed defendants. The decision was made to avoid the additional influence of ethnicity on perceptions of guilt since previous work has demonstrated its salience as a source of bias (Blair et al., 2004; Cothran et al., 2017). Of course, it is possible that different patterns of influence due to attractiveness may be evident when considering other ethnicities, and indeed female defendants (Ahola et al., 2009; Mazzella & Feingold, 1994; Winters et al., 2022), and so future research might focus on addressing these demographics. Related, our participant sample comprised a majority of White individuals, as well as 68% women. This meant that any race biases due to perceiving defendants of another race to one’s own (Mitchell et al., 2005) were minimised, but further work might consider varying the ethnicities of both the participants and the defendants to investigate race in particular. Similarly, the current study was not designed to investigate participant gender, which could play a role in attractiveness biases (e.g., Wuensch et al., 1991), and so this factor might also be the focus of future studies.

Given the evidence here, and more broadly across the literature, that attractiveness perceptions may bias judgements regarding guilt, it is important to consider whether such biases are unavoidable. We know that forming first impressions through viewing faces is inescapable (Ritchie et al., 2017) and that these can be difficult to alter subsequently (Goller et al., 2018). Indeed, Wetzel and colleagues (1981) demonstrated that participants who were informed about the halo effect, and instructed not to show it, were still very susceptible to its effects in their judgements. In a recent study by Jaeger and colleagues (2020), participants were biased by the facial trustworthiness of simulated defendants (known to be strongly correlated with attractiveness; Oosterhof & Todorov, 2008) when making decisions regarding guilt, as well as the amount of damages awarded to the plaintiff. Importantly, when a new sample of participants was educated about the biasing effects of facial stereotypes, the influence of perceived trustworthiness was not reduced, demonstrating its persistence despite this intervention. As such, it seems unlikely that any biases resulting from the perceived attractiveness of defendants can be easily extinguished, and this remains an important avenue for future research.

In conclusion, the current study takes steps towards improving the ecological validity of previous work by utilising short video clips of simulated defendants, real-world descriptions of crimes, and a range of faces varying in attractiveness. While no attractiveness bias was evident for crimes involving burglary, our results suggested that perceptions of guilt for attractive men were higher when committing sexual assault but lower when committing murder. However, our evidence was not conclusive, although from the perspective of estimating likely effects, we have shown the probable direction for these biases. Importantly, such effects were much smaller than initially anticipated.
